# A review of the leishmanin skin test: A neglected test for a neglected disease

**DOI:** 10.1371/journal.pntd.0009531

**Published:** 2021-07-22

**Authors:** Jessica Carstens-Kass, Kayla Paulini, Patrick Lypaczewski, Greg Matlashewski

**Affiliations:** Department of Microbiology and Immunology, McGill University, Montreal, Canada; Institut Pasteur de Tunis, TUNISIA

## Abstract

The leishmanin skin test (LST) has been used for decades to detect exposure and immunity to the parasite *Leishmania*, the causative agent of the neglected tropical disease leishmaniasis. In the LST, *Leishmania* antigen (leishmanin) is intradermally injected into the forearm. In an individual who has been previously infected, a delayed-type hypersensitivity (DTH) reaction results in a measurable induration at the site of the injection, indicating that previous exposure to *Leishmania* has resulted in the development of cell-mediated immunity. LST positivity is associated with long-lasting protective immunity against reinfection, most notably as reported for visceral leishmaniasis (VL). Despite efforts over the past few decades, leishmanin antigen is no longer produced under good manufacturing practice (GMP) conditions anywhere in the world. Consequently, the use of the LST in epidemiological studies has declined in favor of serological and molecular tests. In this review, we provide a historical overview of the LST and justification for the reintroduction of leishmanin. A GMP-grade leishmanin can be used to detect immunity in vivo by the LST and can be investigated for use in an interferon-γ release assay (IGRA), which may serve as an in vitro version of the LST. The LST will be a valuable tool for surveillance and epidemiological studies in support of the VL elimination programs and as a surrogate marker of immunity in vaccine clinical trials.

**Methods:**

A review of the literature was conducted using PubMed as the primary database, with MeSH terms “leishmanin skin test” OR “Montenegro test” OR “Montenegro skin test.” Articles written in English that describe the history or standardization of leishmanin, the use of leishmanin in an IGRA, or the use of the LST in epidemiological studies or vaccine trials were prioritized in our appraisal of the literature.

## Introduction

Leishmaniasis is caused by intracellular protozoan parasites of the genus *Leishmania*, which are transmitted by sand flies in endemic regions of Asia, Africa, Southern Europe, and Latin America [1]. More than 1 billion people are at risk of infection, and up to 1 million new cases occur annually [2]. Leishmaniasis is the second most deadly parasitic disease after malaria and is classified by the World Health Organization as a neglected tropical disease [3]. Depending on the species of *Leishmania* one becomes infected with, the severity of the disease can range from a self-healing cutaneous infection to a life-threatening visceral infection (Table 1) [1]. Cutaneous leishmaniasis (CL) is characterized by nodules and ulcers—usually on the face, arms, and/or legs—which usually self-heal within 3 to 18 months but leave scars, which can be stigmatizing [1,2]. Up to 10% of CL cases progress to leishmaniasis recidivans, diffuse CL, disseminated CL, or mucocutaneous leishmaniasis [1]. In cases of mucocutaneous leishmaniasis, the most disabling form of CL, the infection destroys the mucosal surfaces of the nose, mouth, and throat, leading to disfigurement, stigmatization, and, in some cases, death [2]. Visceral leishmaniasis (VL), or kala-azar, is a systemic manifestation of the disease and is the most severe. VL causes fever, weight loss, anemia, immunosuppression, and enlargement of the spleen and liver and is fatal unless treated [1,2]. Following cure of VL from *Leishmania donovani* infection, patients may develop post-kala-azar dermal leishmaniasis (PKDL), which presents as a rash caused by an immune response against residual parasites in the skin [1]. PKDL is usually self-healing in Africa but is rarely self-healing in the Indian subcontinent [4,5]. Although PKDL is most often an esthetic problem, it can be debilitating in some patients [1] and is thought to be a reservoir of the disease [6,7].

**Table 1:** Clinical and epidemiological characteristics of selected *Leishmania* species.

	Clinical form	High-burden countries or regions
*Leishmania donovani*	VL and PKDL	India, Bangladesh, Ethiopia, Sudan, and South Sudan
*Leishmania major*	CL	Iran, Saudi Arabia, North Africa, the Middle East, Central Asia, and West Africa
*Leishmania infantum*	VL and CL	China, Southern Europe, Brazil, and South America for VL and CL; Central America for CL
*Leishmania mexicana*	CL, DCL, and DsCL	South America
*Leishmania amazonensis*	CL, DCL, and DsCL	South America
*Leishmania braziliensis*	CL, DCL, LR, and MCL	South America
*Leishmania guyanensis*	CL, DsCL, and MCL	South America

Adapted from Burza et al. (2018) [1].

CL, cutaneous leishmaniasis; DCL, diffuse cutaneous leishmaniasis; DsCL, disseminated cutaneous leishmaniasis; LR, leishmaniasis recidivans; MCL, mucocutaneous leishmaniasis; PKDL, post-kala-azar dermal leishmaniasis; VL, visceral leishmaniasis.

The leishmanin skin test (LST; otherwise known as the Montenegro skin test) is performed via intradermal injection of *Leishmania* antigens (leishmanin) to induce and visualize the adaptive immune response in individuals who have been previously infected with *Leishmania*. The test is analogous to the better-known Mantoux tuberculin skin test (TST) [8], which is widely used as a diagnostic test for tuberculosis. Both the LST and TST are based on the principle that the injection of antigens into an individual who has been previously infected causes the classical T cell–mediated inflammatory reaction known as the delayed-type hypersensitivity (DTH) response. In this response, antigen-specific T_H_1 cells are activated by antigen-presenting cells, causing the T_H_1 cells to secrete the inflammatory cytokine interferon-γ (IFN-γ), which recruits macrophages and other inflammatory cells to the site of the injection, resulting in a visible induration [9]. The DTH response is called “delayed” because the maximal influx of T_H_1 cells and other inflammatory cells occurs 24 to 72 hours after exposure to antigens [10]; thus, this is the time at which the size of the induration peaks and is measured. In the case of the LST, an induration of at least 5 mm in diameter at 48 hours is typically considered a positive test [11], indicating that previous exposure to *Leishmania* has resulted in the development of cell-mediated immunity. Since the protective immune response to *Leishmania* is primarily mediated by T_H_1 cells [12], a positive LST result signifies a level of immunity against the disease.

The LST has been a useful tool in epidemiological studies to monitor exposure and immunity to *Leishmania* as well as in vaccine studies as a surrogate marker of immunity. However, a standardized and reliable leishmanin product is currently not available; thus, the use of the LST has recently declined. In this review, we present the history of the LST and call for developing a standardized leishmanin that is produced under good manufacturing practice (GMP) conditions and obtains the World Health Organization diagnostic prequalification for use in endemic countries.

## History of the leishmanin skin test

### Development of the leishmanin skin test

The LST was introduced in 1923 when it was demonstrated that intradermal injection of *Leishmania* extracts into an immunized guinea pig results in a cutaneous reaction [13]. This result was then reproduced in humans by João Montenegro, for whom the test is named, who showed that the reaction is positive in patients with active CL in a sensitive and specific manner [13]. It was soon demonstrated that the LST can detect cellular immunity even in cases in which parasites are too scarce to be found by microscopy in biological samples; thus, the LST became the preferred diagnostic tool for CL [14]. In 1966, it was observed that the LST is often positive in individuals lacking scars to indicate previous infection and who do not recall ever having had symptoms, and it was suggested that these individuals had asymptomatic infections [15]. This observation has since been repeated [16,17], and it is now believed that of all people infected with *Leishmania*, symptomatic cases represent merely the tip of the iceberg. Although the LST was historically used to help diagnose skin lesions as cases of active CL, its use as a diagnostic tool is questionable due to the fact that the test does not distinguish between active and cured CL. Thus, use of the LST for diagnosis has declined since the emergence of alternative diagnostic tools that detect *Leishmania* DNA [18,19]. However, the LST is currently still used in epidemiological studies of CL and is often recommended as a complimentary diagnostic test in endemic regions in South America [8,20].

The application of the LST to VL was more complex. In 1944, it was reported that the LST is negative in patients with active VL [21]. This was believed to be due to cellular anergy [1] and eliminated the possibility of using the LST as a diagnostic tool for VL. At the time, this finding led to the belief that the skin retains a sensitivity to leishmanin following CL but not VL. Therefore, it was believed that the LST was only useful to CL. Fifteen years later, it was hypothesized that the LST would be positive in cases of PKDL due to the presence of parasites in the skin [21]. Surprisingly, it was shown that the LST is positive once VL is cured regardless of whether the patient develops PKDL [21], unleashing a wave of new possible applications of the LST in the context of cured VL. Furthermore, it was quickly demonstrated that, like in CL, the LST is positive in individuals with asymptomatic VL infections, and it was proposed that these asymptomatic cases are reservoirs of *Leishmania* and perpetuate its transmission undetected [22]. This idea led to the first suggestion that the LST be used for surveillance of VL in endemic regions [22]. Since then, the LST has been used for monitoring exposure to VL-causing *Leishmania* species in epidemiological studies [23–26].

### Past sources of leishmanin

When Montenegro first demonstrated the use of the LST in humans, he warned against accepting the results of the test without first evaluating the antigen preparation [13], highlighting the need for a standardized leishmanin. Still, despite repeated attempts at standardization and distribution, there is currently no standardized GMP-grade leishmanin available to researchers or clinicians. In many of the earliest studies, leishmanin was locally produced using varying techniques to obtain a suspension of killed whole promastigotes. The result was that studies used leishmanin products of differing composition and variable quality; thus, these studies could not be compared for analysis [27]. The Wellcome Trust resolved this issue when it began producing the first standardized leishmanin [28] (date unknown). However, production ended in the early 1980s, again forcing researchers either to prepare their own leishmanin or to stop using the LST. In the early 1990s, another attempt at standardization was made when the Special Programme for Research and Training in Tropical Diseases (TDR) at the World Health Organization asked for leishmanin submissions from institutions around the world [28]. The 3 submissions received were a *Leishmania amazonensis*–derived leishmanin from the University of Minas Gerais in Brazil, a *Leishmania infantum*–derived leishmanin from the Istituto Superiore de Sanità in Italy, and a *Leishmania major*–derived leishmanin from the Pasteur Institute in Iran [28,29]. The latter 2 products were found to be more potent, and TDR selected the Iranian leishmanin to support a developing country [28]. TDR began wide distribution of the Iranian leishmanin, resolving the need for a standardized and reliable antigen [27,28]. TDR eventually ceased the distribution of the Iranian leishmanin for unknown reasons. However, some institutions, including those mentioned above, briefly continued to produce leishmanin on a smaller scale. The Iranian leishmanin was still used in studies until recently [30,31], but it is no longer available today (personal communication, Professor Alimohammadian). The leishmanin from the Istituto Superiore de Sanità was produced until a study suggested that the sensitivity of the product declines over time [29]. The Centro de Produção e Pesquisa de Imunobiológicos (CPPI, Immunobiology Production and Research Center) was the last company producing the Brazilian leishmanin; production of the Brazilian leishmanin ended in 2017 when the Agência Nacional de Vigilância Sanitária (ANVISA, National Health Surveillance Agency), a regulatory agency belonging to the Brazilian Ministry of Health, redefined its requirements for the manufacturing of the reagent [32]. The Wellcome Trust also resumed its production of leishmanin for a time, but eventually stopped once again. Consequently, researchers today have no source of standardized leishmanin.

### Production, storage, and use of leishmanin: What can be learned from the tuberculin skin test?

Few studies have compared the various methods of production, storage, and use of leishmanin, which is important for ensuring that studies using leishmanin are reliable and comparable. In this section, we will present the various techniques that have been employed in these 3 areas. When possible, we will attempt to identify which methods are optimal. Since tuberculin is standardized, extensively studied, and widely used, it is often a helpful benchmark for comparison.

### Production and storage of leishmanin

Cross-reactivity between *Leishmania* species has been observed [33–35], although the extent of such cross-reactivity is unknown. While more studies are needed to elucidate the level of cross-reactivity between *Leishmania* species in the LST, there does appear to be a consensus that cross-reactivity is strongest within CL-causing and VL-causing subsets of *Leishmania* species, respectively [35,36]. Although it would be difficult to make multiple versions of leishmanin for different endemic regions, for maximum sensitivity, it may be useful to make an *L*. *donovani*–derived leishmanin for VL and a leishmanin derived from an Old World and a New World species for CL.

While leishmanin was originally made of killed whole promastigotes, it has since been reported that soluble *Leishmania* antigen is more sensitive and potent [27,37]. The preparation of soluble leishmanin involves the disruption of parasites, and it has been suggested that this process causes immunogenic epitopes to be more available for uptake by antigen-presenting cells, resulting in the increased sensitivity [27]. There are further advantages of using soluble antigen rather than killed whole promastigotes: It can be sterilized by filtration; the protein content can be determined and standardized; and the homogeneity of the solution allows it to be delivered with greater uniformity [38]. However, the preparation of soluble leishmanin is inconsistent across studies. To begin preparing leishmanin, harvested parasites are centrifuged, washed, and the pellet is then resuspended. The media in which the pellet is resuspended is a source of discordance between studies. A few examples of media that have been used are sterile water [27,37]; phenol solution [8]; saline solution containing 0.0001% thimerosal (merthiolate) [39]; and Tris-HCl, EDTA, leupeptin, α-2-macroglobulin, and phenylmethylsulfonyl fluoride [40]. Once the pellet has been resuspended, the next step is the disruption of the parasites, which has been accomplished in these studies by freeze-thaw cycles [27,37], sonication [39,40], or both. The disrupted parasite lysate is clarified by centrifugation, and the supernatant is collected. Sometimes, this supernatant is further dialyzed [40] or sterilized by filtration through 0.22-μm pores [8,27,40]. These various techniques should be compared to establish consistency in the production of soluble leishmanin. Once the methodology has been established, it will be essential to manufacture leishmanin under GMP certification, which ensures the integrity of the manufacturing process and compliance with safety regulations. GMP-grade leishmanin must then be validated for safety and sensitivity in previously infected and relevant control populations.

The volume of leishmanin injected during the LST is consistently 0.1 ml in previous studies, but the amount of leishmanin protein delivered by the injection is often inconsistent due to the different preparation techniques described above. The sensitivity of leishmanin plateaus near 100% as dose increases [37,41]. Also, size of induration increases as dose increases [27,42]. However, very high doses of leishmanin have been associated with vesiculation, ulceration, and necrosis, which may lead to false-positive readings [42]. This underlines the need to deliver an optimal dose of leishmanin in the LST. The first study using soluble leishmanin reported that 25 μg was the better dose compared to 5 μg and 50 μg [37], and a recent study using the LST delivered a 25-μg dose [43].

To prevent its degradation during storage, leishmanin should be prepared with preservatives. Phenol prevents contamination but reportedly does not stabilize proteins [42], while the surfactant polysorbate 80 serves as a stabilizer as well as an excipient [44]. In tuberculin, a concentration of 0.0005% polysorbate 80 is sufficient to prevent adsorption of proteins, especially to the inner walls of glass or plastic containers, helping to maintain potency for skin testing [45]. Tuberculin prepared with phenol (0.3%) and polysorbate 80 (0.0005%) has been found to be stable following up to 3 years of storage at 4°C [46]. Accordingly, the first soluble leishmanin contained phenol (0.28%) and polysorbate 80 (0.0005%) [37]. Presumably, phenol and polysorbate 80 have the same preservative effects on leishmanin as they do on tuberculin.

The temperature at which leishmanin is stored also affects its stability. In the field, temperature control may be spotty or unavailable during transportation and storage. It is therefore necessary for leishmanin to be stable when stored refrigerated as a liquid. Even more ideal would be for leishmanin to be stable at the high ambient temperatures of endemic regions, which, for example, reach 29°C to 38°C during summer months in the Middle East [47]. Tuberculin is stored in liquid form at 2°C to 8°C [48]. However, soluble leishmanin containing phenol (0.28%, w/v) and polysorbate 80 (0.0005%, w/v) stored at 2°C to 8°C for 5 years displayed significant protein degradation [38]. Additionally, lyophilization (freeze drying) extends the shelf life of tuberculin [49] and increases the heat stability of the bacille Calmette–Guérin (BCG) vaccine for tuberculosis [50], but lyophilization has not been explored as an option for leishmanin. Studies could be performed in experimental animal models such as guinea pigs [51] to compare the stability and immunogenicity of liquid or lyophilized soluble leishmanin stored at various temperatures over assorted time periods.

### Standardization of the LST

Once a standardized soluble leishmanin is produced, the LST itself should be standardized. Studies using the LST have measured induration at different time points ranging from 48 to 72 hours after injection of leishmanin [27,52–54]. If only one reading is possible, it would be ideal to measure induration 48 hours after the injection of leishmanin, as this is the time at which peak induration is most likely to occur for skin tests in general [55]. In a study of the LST in 2,500 participants, indurations were largest 48 hours after the injection of leishmanin but were not statistically significant in size from indurations measured 72 hours after the injection of leishmanin [56]. Future studies should take into consideration the kinetics of the cellular immune response in the LST.

**Fig 1:**
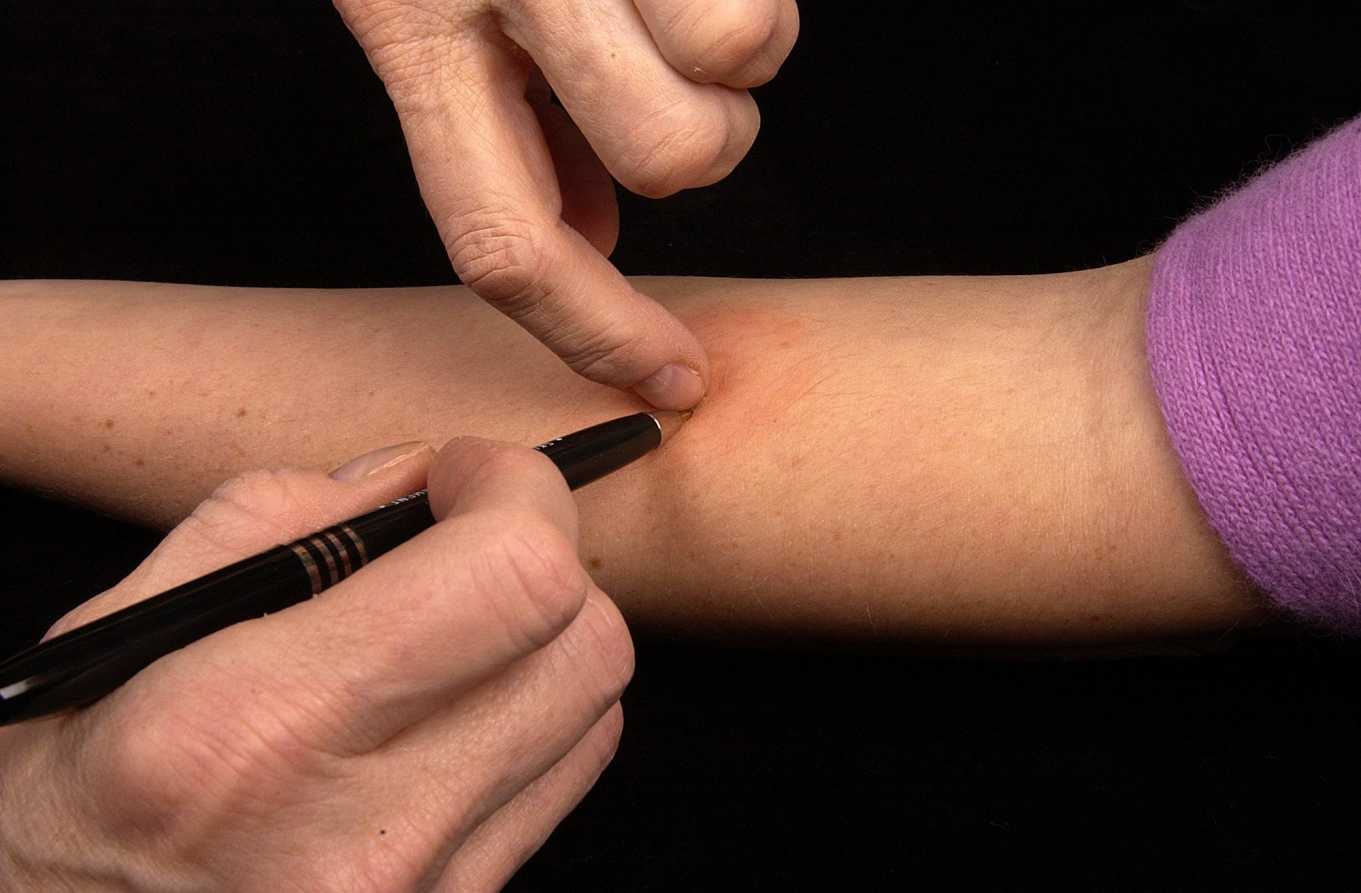
Ballpoint pen method for reading transverse diameter of induration. A ballpoint pen is placed a few centimeters from the edge of the induration and lightly moved across the skin toward the induration, drawing a line on the skin. When the tip of the pen reaches the boundary of the induration, added pressure is felt by the hand holding the pen, at which point the pen is lifted from the skin. Thus, the end of the line marks a boundary of the induration. Image from CDC Public Health Image Library [58].

Various studies have used different measurements and calculations to determine the size of an induration: One diameter [20]; the mean of 2 perpendicular diameters [27,30,39,57] except in cases of highly asymmetric reactions [10]; and the mean of the longest diameter and its perpendicular [42] have all been used. Additionally, there is reader-to-reader variability in skin tests, mainly due to the fact that induration boundaries can be difficult to define [55]. This issue can be somewhat resolved within studies by the consistent use of a single experienced reader [55] and across studies by clearly defining induration boundaries using the ballpoint pen method [10,58] (Fig 1). The ballpoint pen method is widely used in the TST [59].

### Advantages of LST over serology for epidemiological studies

The goal of many epidemiological studies of leishmaniasis is to track and monitor the exposure of a certain population to *Leishmania*. Since their development, serological assays such as the direct agglutination test or the enzyme-linked immunosorbent assay (ELISA) have often been used for this purpose [60]. There are, however, 2 reasons why the LST is advantageous over serology for determining exposure to *Leishmania*. First, cell-mediated immunity lasts longer than antibody-mediated immunity following cured leishmaniasis [61]. In the case of CL, parasites are confined to the skin and therefore elicit a predominantly cell-mediated immune response [62]. This explains why antibody titres are low during and following even severe CL infection [62,63]. Thus, the LST would be more effective than serology at determining past exposure to CL-causing *Leishmania* species. In the case of VL, LST positivity can last anywhere from 20 years to a lifetime [17,23], while humoral immunity nearly disappears by the completion of treatment [64]. In many populations residing in CL- and VL-endemic regions, LST positivity trends upward with age while seropositivity does not [25,30,31,52,56]. It is unclear whether LST positivity increases with age due to naturally long-lasting cellular immunity or increased cumulative exposure to *Leishmania* or both. Nevertheless, this illustrates the longevity of cell-mediated immunity and supports the claim that the LST is a better indicator than serology of past exposure to both CL- and VL-causing *Leishmania* species. Accordingly, studies conducted around the world have found that the LST is more sensitive than serology [52,65,66].

Second, in the case of VL, cell-mediated immunity is more indicative of protection against reinfection compared to antibody-mediated immunity [57]. LST–positive individuals are largely immunologically protected against VL [25,26,29,67] and are less likely to be reinfected compared to seropositive individuals [25]. Conversely, asymptomatic seropositive individuals in Bihar, India are considered to be at higher risk of developing VL [68]. With respect to New World CL, the size of induration produced by the LST-induced DTH response is negatively correlated with probability of reinfection, as incidence decreases by 17.9% per mm [56]. Thus, not only does a positive LST response indicate protection against reinfection but may also indicate the degree of protection.

### LST in vaccine trials

Promising new vaccines for leishmaniasis are emerging [69,70]. A major challenge for developing a vaccine for leishmaniasis will be how to measure efficacy. Development of disease has been traditionally used as a primary endpoint in human vaccine studies [71]. However, endpoint efficacy trials will be difficult to conduct in areas where there are fewer than 5 cases per 10,000 persons due to the success of ongoing VL elimination programs [72]. Therefore, a surrogate marker of immunity is needed to determine vaccine efficacy. As discussed above, LST positivity following natural infection is associated with long-lasting protection against VL [25,26,29,67]. LST conversion has been used as an endpoint for protective immunity in trials involving first-generation vaccines with heat-killed parasites in the Old World [73] and in the New World [74,75]. Overall, since these vaccines were not protective and poor at producing a positive LST, it was not possible to firmly conclude that a positive LST was associated with protective immunity. Reintroducing the LST with a standardized leishmanin will nevertheless be required as a surrogate marker of protective immunity for future trials with the newer and potentially more effective vaccine candidates [69,70]. The live-attenuated vaccine that is advancing toward human clinical trials [70] provides an excellent opportunity to make leishmanin under GMP conditions.

For vaccine trials, LST negativity can be used to identify individuals who have not been previously exposed to *Leishmania* and therefore are good candidates for the trial. However, there is concern that leishmanin itself has a sensitizing effect, meaning an LST could cause positivity in subsequent LSTs resulting in a false-positive interpretation of vaccine efficacy. There is some evidence that the LST does not sensitize persons to leishmanin when leishmanin is prepared from whole promastigotes [42,76]. However, repeated LSTs using a leishmanin prepared from whole promastigotes of a mixture of *L*. *amazonensis*, *Leishmania braziliensis*, and *Leishmania guyanensis* was reported to be sensitizing [77]. Once a standardized soluble leishmanin is prepared, it should be tested for sensitizing effects. If soluble leishmanin is not sensitizing, then the LST may be used prior to vaccination to determine previous exposure and again after vaccination. If soluble leishmanin is sensitizing, then the LST should only be used after vaccination to determine the vaccine-induced immune response. In the latter case, an in vitro version of the LST could be used prior to vaccination to determine previous exposure, as discussed in the following section.

## Potential application of leishmanin to the interferon-γ release assay

The development of monoclonal antibodies against IFN-γ in 1990 [78] made it possible to design a sandwich ELISA to detect IFN-γ in the supernatants of whole blood samples stimulated with tuberculin (also referred to as purified protein derivative or PPD) [79]. This was the first IFN-γ release assay (IGRA), developed to diagnose bovine tuberculosis and later used to diagnose human tuberculosis. The cell-mediated immune response against *Mycobacterium tuberculosis* is driven by the activation of macrophages by IFN-γ [80]. An IGRA detects whether immune cells increase production of IFN-γ in response to stimulation with a particular antigen; an increase in IFN-γ production indicates the presence of antigen-specific cell-mediated immunity. Thus, like a skin test, the IGRA shows whether an individual has cell-mediated immunity against the antigens of a specific pathogen, and this could have advantages for some leishmaniasis applications.

The IGRA is quick, easy to perform, and more sensitive and specific than any other tests available for the diagnosis of bovine and human tuberculosis, including the TST [79,81,82]. Thus, the IGRA emerged as a new diagnostic tool for human tuberculosis and a commercially produced IGRA called QuantiFERON was subsequently developed. To perform the QuantiFERON test, 1 ml of blood is collected directly in QuantiFERON tubes in the field and transported to a laboratory testing facility. Within 24 hours, tuberculin antigens are added to the tubes, and the IFN-γ concentration of the supernatants is measured using a 1-step ELISA kit [83]. Field use is possible because the only components required are the QuantiFERON tubes (with *Leishmania* antigen) and a hot water bath to incubate at 37°C. In 2001, the Food and Drug Administration (FDA) approved a commercial QuantiFERON-TB test [84], providing researchers with a standardized tool with which to diagnose tuberculosis in vitro in the field [35]. Since then, the QuantiFERON and updated versions have been widely used for diagnosis and in epidemiological studies of tuberculosis.

Similar to the immune response against *M*. *tuberculosis*, the protective immune response against *Leishmania* is cell mediated and relies on the activation of macrophages by IFN-γ [12]. In 2010, in response to the lack of standardization of leishmanin for use in the LST, a modified QuantiFERON was investigated as an alternative to the LST for epidemiological studies [85]. Tuberculin antigens were replaced with *Leishmania* peptide antigens in the QuantiFERON-TB Gold In-Tube test. Whole blood stimulated with specific *Leishmania* peptides released IFN-γ in a sensitive and specific manner. A similar study showed that the test has even higher sensitivity when soluble leishmanin is used for stimulation instead of *Leishmania* peptides [83]. When this study was repeated with a larger participant pool, the modified QuantiFERON using soluble leishmanin for stimulation was 85% sensitive and 100% specific and detected asymptomatic, active, and cured cases of VL [86]. These findings illustrate that the IGRA could be an additional application for soluble leishmanin, providing yet another compelling reason to develop a standardized leishmanin product.

The level of concordance between the LST and modified QuantiFERON has been evaluated in studies that measured the levels of IFN-γ released by stimulated peripheral blood mononuclear cells [35,87]; however, studies are needed which evaluate the levels of IFN-γ released by stimulated whole blood, as this is a better reflection of disease status for VL for reasons that are currently unknown [86,88]. A study should be conducted in which the LST and the IGRA are performed on the same cohort to determine concordance between the modern versions of these tests. The results will help in determining whether it is appropriate to replace the LST with the modified QuantiFERON in certain scenarios, such as prior to vaccination trials. Regardless of which test is chosen in future studies, researchers are in need of a standardized soluble leishmanin product.

## Future directions

In this review, we have discussed the past uses of the LST, the motivation for reintroducing the LST, and the need for a standardized leishmanin product. We will now consider how the LST may be applied once a standardized leishmanin product is available.

An urgent application of the LST is to support the effort to eliminate VL in the Indian subcontinent. Elimination of VL from this region has been deemed achievable for the following reasons [89]: *L*. *donovani*, the causative species of VL in the region, has no nonhuman reservoirs; *Phlebotomus argentipes*, the sole vector of *L*. *donovani* in the region, is susceptible to insecticides; transmission is geographically restricted to a focused area; there are effective options for diagnosis (rk39 rapid diagnostic test [90]) and treatment (oral miltefosine and liposomal amphotericin B) [1]; and immunity is protective and long lasting. In 2005, Bangladesh, India, and Nepal launched a 10-year initiative to eliminate VL with a target of less than 1 case per 10,000 at the block level [91]. This initiative contributed to an impressive 75% decline in incidence by 2015 [92]. However, the countries have yet to achieve sustainable elimination in 2020, with India being the furthest from the target [93]. A lack of effective surveillance has been a persistent constraint since the launch of the initiative [94,95]. Specifically, studies are needed to understand why VL tends to cluster in shifting and tightly localized areas and how the parasite reservoir is maintained in locations where there are few VL cases [96]. The LST could be used to conduct epidemiological studies to establish the levels of ongoing and previous transmission in districts with varying numbers of cases. For these studies, it may be necessary to consider using a leishmanin derived from *L*. *donovani*, the species endemic in these regions, to increase sensitivity of the LST. A high level of LST positivity, particularly in the young, would identify areas of active transmission where increased resources such as active case detection and vector control efforts could be focused.

The development of a vaccine would advance the elimination of VL from the Indian subcontinent and Africa as well as help prevent all forms of leishmaniasis worldwide. As promising vaccines advance to clinical trials, it would be valuable to use the LST as a surrogate marker of immunity and protection. This would greatly reduce the number of participants and time required to show vaccine-induced protective immunity, simplifying Phase III trials. The eligibility of participants for vaccine trials should be based on the negative result of a modified QuantiFERON, because this test does not expose study participants to leishmanial antigens. This will ensure that an LST performed after vaccination will not be affected by a participant’s exposure to anything other than the vaccine itself, making the LST a more reliable surrogate marker of immunity.

**Fig 2:**
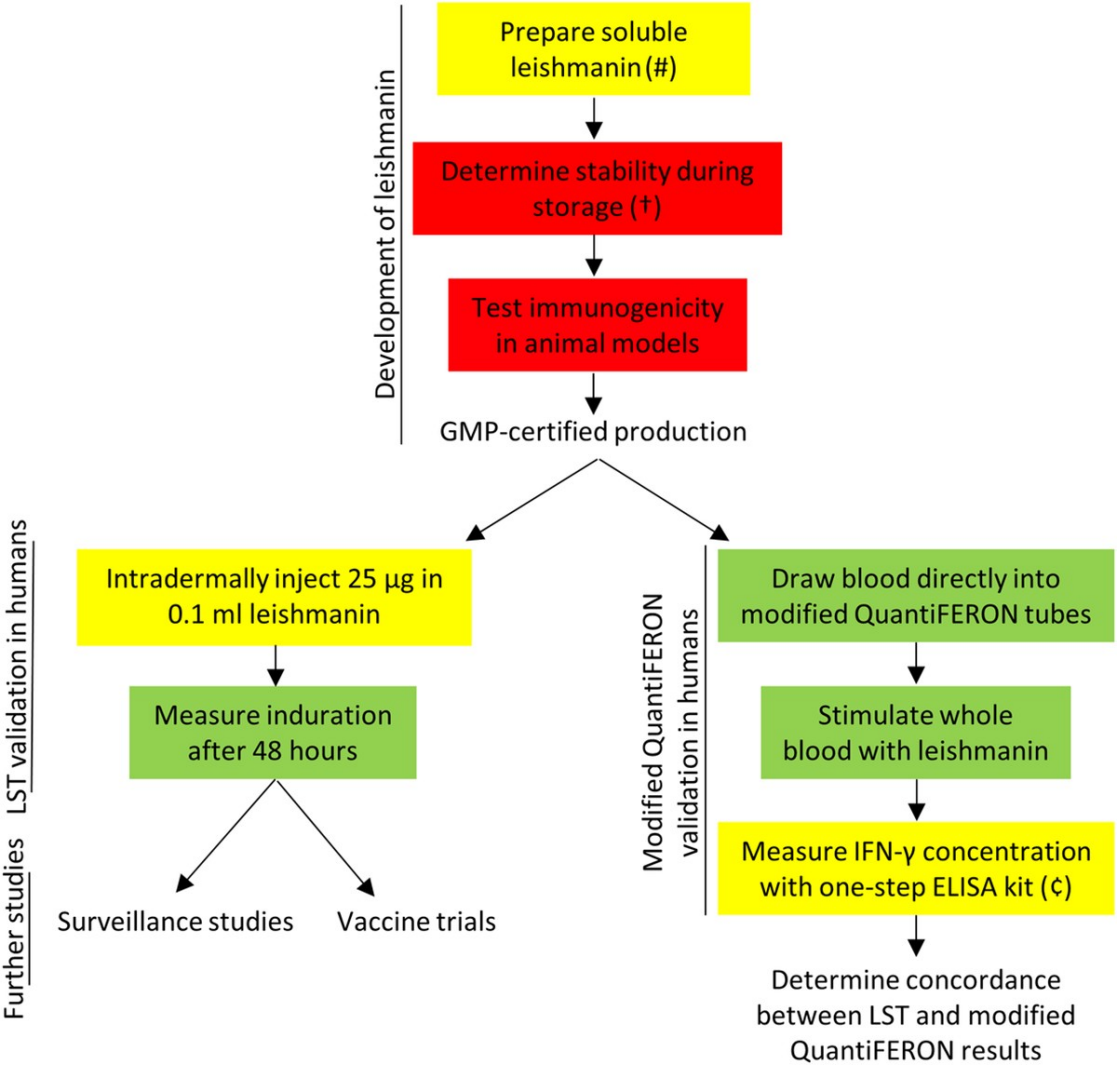
Steps required for the production, validation, and application of leishmanin. Green = method is standardized. Yellow = method requires confirmational studies. Red = method is not standardized. Symbols (#, †, ¥, and ¢) correspond to recommendations in Table 2. ELISA, enzyme-linked immunosorbent assay; GMP, good manufacturing practice; IFN-γ, interferon-γ; LST, leishmanin skin test.

In summary, the LST is an effective surveillance tool for detecting exposure and immunity to *Leishmania*, but a standardized GMP-grade leishmanin antigen is not currently available anywhere in the world. In this review, we have outlined some of the major considerations required to prepare, store, and use leishmanin, as well as the studies required to standardize these steps (Fig 2, Table 2). We have also discussed the benefits of the LST compared to serological tests for epidemiological studies, the use of the LST in vaccine trials, and the potential application of leishmanin to the IGRA. In conclusion, the reintroduction of the LST is urgently needed for ongoing surveillance and elimination programs and future vaccine trials.

**Table 2:** Recommendations for future development and studies.

**Standardization of the production and storage of leishmanin**
Determine cross-reactivity of *Leishmania* species in leishmanin preparations (#)
Establish standardized methodology for production of leishmanin under GMP guidelines (#)
Standardize the use of safe preservatives in leishmanin and determine whether lyophilization of leishmanin improves storage and heat stability (†)
**Administration of the LST**
Standardize the metric used to measure size of induration (¥)
**Future applications of leishmanin and the LST**
Use the LST alone or in combination with serological assays for surveillance and epidemiological studies
Use the LST for the diagnosis of CL
Use the LST as a surrogate marker of immunity in vaccine trials
Use leishmanin in the modified QuantiFERON (an IGRA)
Determine the best positive reference value for the modified QuantiFERON (¢)
Determine the level of concordance between the LST and modified QuantiFERON

Symbols (#, †, ¥, and ¢) correspond to steps in Fig 2.

CL, cutaneous leishmaniasis; IGRA, interferon-γ release assay; LST, leishmanin skin test.

**Key Learning Points**

- The leishmanin skin test (LST) has been used for almost 1 century to detect exposure and immunity to *Leishmania* parasites, which cause the disease leishmaniasis.
- The LST is better than serological tests for detecting exposure and immunity to *Leishmania* and can be used for epidemiological studies, surveillance, and as a surrogate marker of protective immunity for human vaccine trials.
- *Leishmania* antigen (leishmanin), the reagent used in the LST, is not currently produced under good manufacturing practice (GMP) conditions anywhere in the world. As a result, the LST is seldom used in the field today.
- A standardized leishmanin is defined as an antigen that is produced under GMP conditions and obtains the World Health Organization prequalification.
- A standardized leishmanin product can also be investigated for use in an interferon-γ release assay (IGRA), which may serve as an in vitro version of the LST.

**Top Five Papers**

1. Gidwani K, Jones S, Kumar R, Boelaert M, Sundar S. Interferon-gamma release assay (modified QuantiFERON) as a potential marker of infection for *Leishmania donovani*, a proof of concept study. PLoS Negl Trop Dis. 2011 Apr 19;5(4):e1042.
2. Montenegro J. Cutaneous reactions in leishmaniasis. Arch Derm Syphilol. 1926 Feb 1;13(2):187–94.
3. Reed SG, Badaró R, Masur H, Carvalho EM, Lorenco R, Lisboa A et al. Selection of a skin test antigen for American visceral leishmaniasis. Am J Trop Med Hyg. 1986 Jan 1;35(1):79–85.
4. Weigle KA, Valderrama L, Arias AL, Santrich C, Saravia NG. Leishmanin skin test standardization and evaluation of safety, dose, storage, longevity of reaction and sensitization. Am J Trop Med Hyg. 1991 Mar 1;44(3):260–71.
5. Zijlstra EE, El-Hassan AM, Ismael A, Ghalib HW. Endemic kala-azar in eastern Sudan: a longitudinal study on the incidence of clinical and subclinical infection and post-kala-azar dermal leishmaniasis. Am J Trop Med Hyg. 1994 Dec 1;51(6):826–36.
